# Quantitative analysis of diffusion-weighted magnetic resonance imaging in malignant breast lesions using different b value combinations

**DOI:** 10.1007/s00330-012-2687-8

**Published:** 2012-10-31

**Authors:** Line B. Nilsen, Anne Fangberget, Oliver Geier, Therese Seierstad

**Affiliations:** 1 Department of Radiation Biology, Norwegian Radium Hospital, Oslo University Hospital, P.O. Box 4959, Nydalen, 0424 Oslo, Norway; 2 Faculty of Medicine, University of Oslo, P.O. Box 1078, Blindern, 0316 Oslo, Norway; 3 Department of Radiology and Nuclear Medicine, Division of Diagnostics and Intervention, Norwegian Radium Hospital, Oslo University Hospital, P.O. Box 4959, Nydalen, 0424 Oslo, Norway; 4 Department of Diagnostic Physics, The Interventional Centre, Division of Diagnostics and Intervention, Oslo University Hospital, P.O. Box 4950, Nydalen, 0424 Oslo, Norway; 5 Department of Health Sciences, Buskerud University College, P.O. Box 7053, 3007 Drammen, Norway

**Keywords:** Apparent diffusion coefficient (ADC), Monoexponential model, Biexponential model, Breast, Diffusion-weighted magnetic resonance imaging

## Abstract

**Objectives:**

To explore how apparent diffusion coefficients (ADCs) in malignant breast lesions are affected by selection of b values in the monoexponential model and to compare ADCs with diffusion coefficients (Ds) obtained from the biexponential model.

**Methods:**

Twenty-four women (mean age 51.3 years) with locally advanced breast cancer were included in this study. Pre-treatment diffusion-weighted magnetic resonance imaging was performed using a 1.5-T system with b values of 0, 50, 100, 250 and 800 s/mm^2^. Thirteen different b value combinations were used to derive individual monoexponential ADC maps. All b values were used in the biexponential model.

**Results:**

Median ADC (including all b values) and D were 1.04 × 10^-3^ mm^2^/s (range 0.82–1.61 × 10^-3^ mm^2^/s) and 0.84 × 10^-3^ mm^2^/s (range 0.17–1.56 × 10^-3^ mm^2^/s), respectively. There was a strong positive correlation between ADCs and Ds. For clinically relevant b value combinations, maximum deviation between ADCs including and excluding low b values (<100 s/mm^2^) was 11.8 %.

**Conclusion:**

Selection of b values strongly affects ADCs of malignant breast lesions. However, by excluding low b values, ADCs approach biexponential Ds, demonstrating that microperfusion influences the diffusion signal. Thus, care should be taken when ADC calculation includes low b values.

***Key Points*:**

• *Diffusion-weighted sequences are increasingly used in breast magnetic resonance imaging*

• *Diffusion-weighting (b) values strongly influence apparent diffusion coefficients of malignant lesions*

• *Exclusion of low b values reduces the apparent diffusion coefficient*

• *Flow-insensitive monoexponential apparent diffusion coefficients approach biexponential diffusion coefficients*

## Introduction

The apparent diffusion coefficient (ADC), derived from non-invasive, in vivo diffusion-weighted magnetic resonance imaging (DW MRI), is increasingly being included as a quantitative parameter in the radiological assessment of cancer [1, 2]. The ADC reflects the Brownian intra- and extracellular motion of water molecules in biological tissue and thus provides information about the tumour microenvironment [3]. Calculation of the ADC requires acquisition of at least two series of DW MR images with different degrees of diffusion-weighting (b value). Increasing b value leads to decreasing signal intensity (SI) on DW MRI images. The SI attenuation can be described by the monoexponential function:

1 $$ SI=S{I_0}\cdot {e^{{-b\cdot ADC}}} $$

where SI_0_ is the DW MR image acquired without diffusion-weighting (b = 0 s/mm^2^). The ADC is the gradient of the straight line fitted to the logarithm of this function. Thus, areas of diffusion restriction appear bright on DW MR images and dark on corresponding ADC maps.

Several potential applications for the ADC in breast cancer have been suggested and studied; including detection, characterisation, differentiation of tumours as well as evaluation of neoadjuvant treatment response [1, 2, 4–8]. ADC has been found to correlate with cell density and studies have reported increasing ADC during neoadjuvant chemotherapy, reflecting reduced cellularity due to cell damage and/or death [6–8]. Currently, the most unambiguous results have been found when ADC is used to differentiate between malignant and benign breast lesions [2]. Including ADC in the characterisation of tumours has been shown to increase the diagnostic accuracy compared with dynamic contrast-enhanced MRI alone [9–11]. However, ADC of malignant and benign breast lesions has been shown to overlap, both within and between different studies, and there is no established cut-off value. Reported ADCs of malignant and benign lesions vary from 0.68 to 1.61 × 10^-3^ mm^2^/s [12, 13] and from 1.35 to 1.77 × 10^-3^ mm^2^/s [14, 15], respectively, whereas previously reported ADCs of normal breast tissue range from 1.51 to 2.09 × 10^-3^ mm^2^/s [16, 17]. Different b value combinations and calculation schemes as well as imaging-dependent factors [18] are anticipated to contribute to the large discrepancy in published ADCs.

In addition to tissue diffusivity, the ADC incorporates the effects of the microcirculatory perfusion of blood (flow), especially at low b values (<100–150 s/mm^2^) [1]. ADCs calculated using only low b values are thus flow-sensitive. To reduce perfusion effects and obtain flow-insensitive ADC or ADC_slow_, it has been recommended to omit low b values from the monoexponential model [1]. It has also been suggested that using a biexponential model, accounting for a vascular compartment in addition to the extravascular extracellular and intracellular compartment, may better reflect “true” diffusion without microperfusion contamination [3]. In the biexponential model, the DW MRI SI attenuation is described by the following function:

2 $$ SI=S{I_0}\left[ {\left( {1-{f_p}} \right)\cdot {e^{{-b\cdot D}}}+{f_p}\cdot {e^{{-b\cdot D*}}}} \right] $$

where D* is the pseudo-diffusion coefficient of the vascular compartment occupying a volume fraction, f_p_, and D is the pure water molecular tissue diffusion coefficient of compartment (1-f_p_) [3].

Traditionally, breast ADCs have been calculated using the monoexponential model. But, there is no consensus on an optimal b value combination and generally b = 0 s/mm^2^ is included. The purpose of this study was to examine how the choice of b values included in the monoexponential model affects calculated ADCs and also to compare these with Ds derived from the biexponential model.

## Materials and methods

### Patients

Twenty-four women diagnosed with locally advanced breast cancer were included in this prospective study. The mean age at time of inclusion was 51.3 years (range 37–72 years). Eighteen lesions were categorised as invasive ductal carcinomas (grade 2, *n* = 7; grade 3, *n* = 8; not determined, *n* = 3) and 6 lesions as invasive lobular carcinomas (grade 2, *n* = 4 and grade 3: *n* = 2). The mean initial clinical tumour size (longest tumour diameter) of the breast lesions was 6.7 cm (range 5–11 cm). Written consent was obtained from all women and the study was approved by the regional ethics committee and the protocol review committee of our institution.

### MR examination

The MR examination was performed as part of the radiological pre-treatment assessment of the extent of the disease. Patients underwent imaging in the prone position using a dedicated phased-array bilateral breast coil (CP Breast array coil, Siemens) on a 1.5-T MR system (ESPREE, Siemens, Erlangen, Germany). To reduce motion artefacts during imaging, cotton pads were put inside the breast coils and care was taken to avoid compression of the breasts.

Diffusion-weighted MRI was carried out using a single-shot spin-echo echo-planar imaging sequence with fat-saturated short T1 inversion recovery and b values of 0, 50, 100, 250 and 800 s/mm^2^ in three orthogonal directions, and the total imaging time was 6:52 min. The following DW MRI imaging parameters were used: repetition time (TR) = 10,300 ms; echo time (TE) = 126 ms; inversion time (TI) = 190 ms; slice thickness = 4 mm; slice gap = 2 mm; number of slices = 26; number of excitations (NEX) = 3; field of view = 360 mm × 195 mm; image matrix = 192 × 104; echo spacing = 0.94 ms; bandwidth = 1,240 Hz/pixel; phase encoding from anterior to posterior. The MR examination also included sagittal turbo spin-echo T1-weighted MRI, axial turbo spin-echo T2-weighted MRI and T1-weighted 3D dynamic contrast-enhanced (DCE) MRI with gadopentetate dimeglumine (Magnevist, Schering, Berlin, Germany) as contrast agent (0.1 mmol/kg body weight). The DCE MRI included acquisition of one pre-enhanced and four enhanced sequences with a time resolution of 85 s. DW MRI was acquired before contrast agent injection.

### Diffusion analysis

A region of interest (ROI) was drawn manually within a solid part of each breast lesion on an ADC map calculated using all b values in the commercially available nICE software package (Nordic NeuroLab, Bergen, Norway). The drawing of the ROI was guided by the native b800 image and the DCE MRI subtraction image (second postcontrast DCE MRI image – precontrast DCE MRI image) from the same anatomical location as the ADC map. The placement of the ROI was verified by an experienced breast radiologist. Further calculations of ADCs, Ds, D*s and f_p_s were performed for all ROIs on a voxel-by-voxel basis using in-house written IDL routines (Interactive Data Language [IDL] version 6.3; Research System, Boulder, CO, USA). In each voxel of the ROI, the DW MRI SI as a function of the b value was fitted to the mono- and biexponential models, using Levenberg–Marquardt least squares minimisation implemented in the commercially available IDL procedure MPFIT (Markwardt CB, presented at the 2008 conference Astronomical Data Analysis Software and Systems XVIII). The goodness of fit between measured SI and fitted curves was evaluated by the Pearson’s correlation coefficient squared (*r* ^2^) and the summed squared residuals of the fit (χ^2^).Voxels where the curve fitting resulted in low *r* ^2^ or high χ^2^ were excluded from further analysis.

The 13 different combinations of b values, B0–B12, used in the monoexponential model are shown in Table 1. B0–B5 are b value combinations with currently clinical relevance. All b values (B0_biexp_) were used in the biexponential model. Statistical analysis was performed on median ADC_BX_s, Ds, D*s and f_p_s of individual breast lesion ROIs.

**Table 1:** The different b value combinations (BXs^a^) explored in the study

BX	b values [s/mm^2^]
B0	0, 50, 100, 250, 800
B1	0, 800
B2	50, 800
B3	50, 100, 250, 800
B4	100, 250, 800
B5	100, 800
B6	250, 800
B7	0, 50
B8	0, 100
B9	0, 250
B10	50, 100
B11	50, 250
B12	100, 250
B0_biexp_	0, 50, 100, 250, 800

^a^ *X* ranges from 0 to 12 for b value combinations used in the monoexponential model, and it is 0_biexp_ for the b value combination used in the biexponential model.

### Statistical analysis

All statistical analysis was performed using SPSS version 18.0 (SPSS, Chicago, IL, USA). Ds and ADCs calculated using the different b value combinations were compared using the non-parametric Wilcoxon signed-rank test. The non-parametric Spearman’s ρ correlation was used to evaluate correlations between Ds and different ADCs. The significance level was set at 5 %.

## Results

**Fig. 1:**
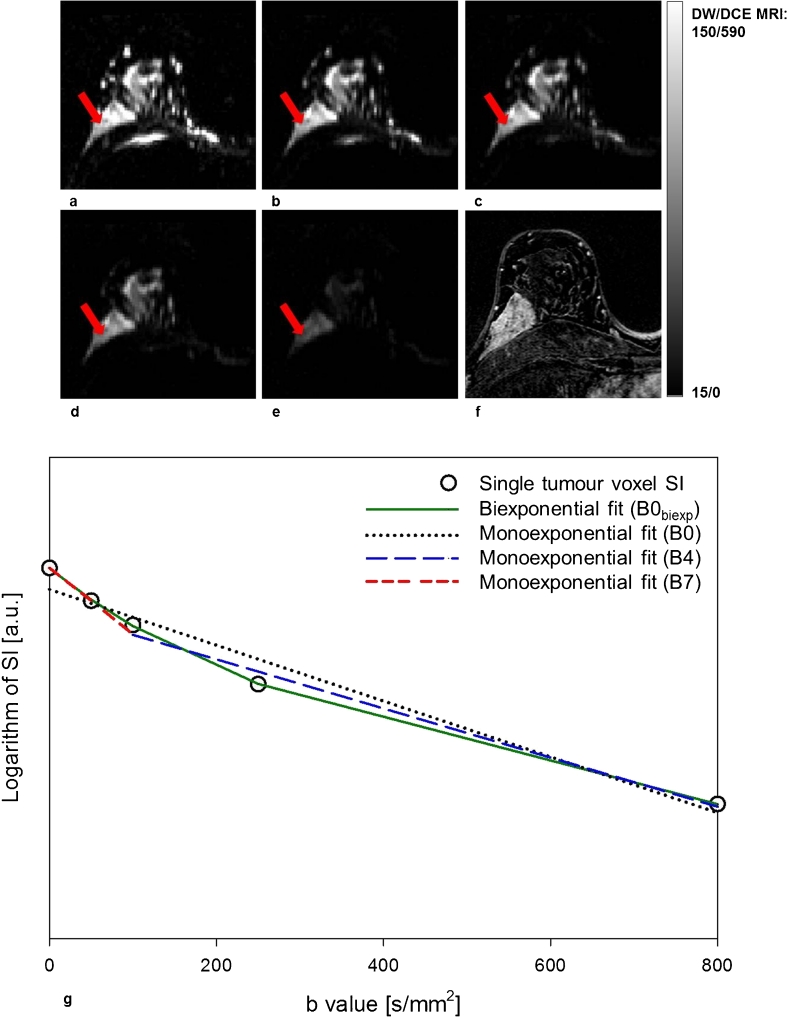
Axial diffusion-weighted images with b values 0 (**a**), 50 (**b**), 100 (**c**), 250 (**d**) and 800 s/mm^2^ (**e**) and corresponding axial contrast-enhanced T1-weighted subtraction image (DCE MRI) (**f**) of the right breast of a patient with invasive ductal carcinoma (grade 2). Attenuation of the DW MRI signal from a single voxel within the breast lesion (*red arrow* in **a**–**e**) is plotted together with the monoexponential and biexponential model fits with all b values (B0/B0_biexp_), b = 100, 250 and 800 s/mm2 (B4) and b = 0 and 50 s/mm^2^ (B7) (**g**)

Twenty-four individual ROIs were drawn and the mean size was 99.2 ± 35.8 mm^2^ (range, 42.2–168.8 mm^2^). All ROI voxels could be fitted to the monoexponential model, whereas on average 79 ± 15 % (range, 37–100 %) of all voxels were satisfactorily fitted to the biexponential model. The results of monoexponential and biexponential fitting with b value combinations B0–B11 are shown in Table 2. Median ADC for the b value combinations using only low b values (B7–B11) were significantly higher than the corresponding ADCs calculated using currently clinically relevant b value combinations (B0-B5) (*P* < 0.001). Figure 1 shows DW MR images for different b values (**a**–**e**) for a woman with a solid contrast-enhancing malignant lesion in the right breast (**f**). Logarithmic SI as a function of b value for a single voxel (*red arrow* in images **a**–**e**) is shown in **g**. Fitted curves using the monoexponential model and all b values (B0, *dotted black line*), only low b values (B7, *dashed red line*) and only high values (B4, *dashed blue line*) are displayed together with the fitted curve obtained using the biexponential model with all b values (B0_biexp_, *solid green line*). The curvature of the SI attenuation is indicative of a non-monoexponential behaviour with increasing b values that is well-characterised by a biexponential function, with an initial drop for b values less than 100 s/mm^2^.

**Table 2:** Comparison of monoexponentially and biexponentially calculated diffusion coefficients: results of calculation with B0–B11 and B0_biexp_

	Mean ± SD [×10^-3^ mm^2^/s]	Median [×10^-3^ mm^2^/s]	Range [×10^-3^ mm^2^/s]	95 % CI [×10^-3^ mm^2^/s]	*r* ^2^
ADC_B0_	1.12 ± 0.24	1.04	0.82–1.61	0.81–1.31	>0.987
ADC_B1_	1.16 ± 0.23	1.09	0.84–1.65		1.0
ADC_B2_	1.10 ± 0.24	1.01	0.78–1.59		1.0
ADC_B3_	1.09 ± 0.24	0.99	0.77–1.58	0.78–1.29	>0.994
ADC_B4_	1.07 ± 0.25	0.97	0.74–1.60	0.53–1.71	>0.997
ADC_B5_	1.08 ± 0.24	0.98	0.76–1.61		1.0
ADC_B6_	1.03 ± 0.25	0.96	0.69–1.64		1.0
ADC_B7_	2.32 ± 0.65	2.24	1.40–3.88		1.0
ADC_B8_	1.89 ± 0.41	1.86	1.11–2.91		1.0
ADC_B9_	1.51 ± 0.30	1.41	1.04–2.18		1.0
ADC_B10_	1.55 ± 0.41	1.56	0.77–2.26		1.0
ADC_B11_	1.32 ± 0.26	1.27	0.98–2.00		1.0
D _B0biexp_	0.90 ± 0.30	0.84	0.17–1.56	0.54–1.22	>0.999

*ADC* monoexponential apparent diffusion coefficient, *D* diffusion coefficient derived from the biexponential model, *B0-B11* and *B0*_*biexp*_ b value combinations defined in Table 1, *SD* standard deviation, *CI* median confidence interval of parameter fit, *r* ^*2*^ Pearson’s correlation coefficient squared

**Fig. 2:**
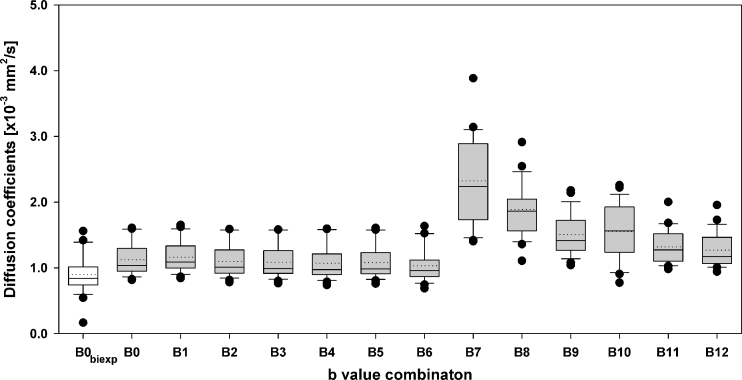
Box-plot of median individual breast lesion diffusion coefficients (*first box in white*) and apparent diffusion coefficients (*grey boxes*) calculated using respectively the biexponential model with all b values (B0_biexp_: b = 0, 50, 100, 250 and 800 s/mm^2^) and the monoexponential model with the following b value combinations: B0 (all b values), B1: b = 0 and 800 s/mm^2^, B2: b = 50 and 800 s/mm^2^, B3: b = 50, 100, 250 and 800 s/mm^2^, B4: b = 100, 250 and 800 s/mm^2^, B5: b = 100 and 800 s/mm^2^, B6: b = 250 and 800 s/mm^2^, B7: b = 0 and 50 s/mm^2^, B8: b = 0 and 100 s/mm^2^, B9: b = 0 and 250 s/mm^2^, B10: b = 50 and 100 s/mm^2^, B11: b = 50 and 250 s/mm^2^ and B12: b = 100 and 250 s/mm^2^. The *horizontal solid and dotted lines* within each box represents the median and the mean, respectively. The *black circles* represent outliers and the *top and bottom of the boxes* show the 95th and the 5th percentiles

Figure 2 shows a box-plot illustrating the distribution of biexponential median Ds (*white box*) and median ADCs (*grey boxes*) for different b value combinations. Comparison of monoexponential median ADC_B0-B5_ and biexponential D is shown in Table 3. Median ADC was reduced by at most 10.6 % (*P* < 0.001) when b values <100 mm^2^/s were excluded from the calculations (Table 3). D was significantly lower than all ADCs_B0–B12_ (*P* < 0.001). Positive correlations were found between Ds and ADCs_B0-B6,B9_ (*P* < 0.01). The largest inter-patient variations were found for b value combinations including b < 100 s/mm^2^ (Fig. 2).

**Table 3:** Comparison of monoexponentially and biexponentially calculated diffusion coefficients: deviation [%] and Spearman’s ρ correlation of median values for B0-B5 and B0_biexp_

	ADC_B0_	ADC_B1_	ADC_B2_	ADC_B3_	ADC_B4_	ADC_B5_	D_B0biexp_
ADC_B0_	0	-4.7***	2.4***	5.1***	6.6***	5.4***	23.2***
ADC_B1_	4.9***	0	7.4***	10.2***	11.8**	10.6***	29.2***
ADC_B2_	-2.3***	-6.9***	0	2.6*	4.1*	3.0*	20.3***
ADC_B3_	-4.8***	-9.3***	-2.5*	0	1.4*	0.3	17.2***
ADC_B4_	-6.2***	-10.6***	-3.9**	-1.4**	0	-1.1*	15.6***
ADC_B5_	-5.1***	-9.6***	-2.9*	-0.3	1.1*	0	16.8***
D_B0biexp_	-18.8***	-22.6***	-16.9***	-14.7***	-13.5**	-14.4***	0
Correlation: D_B0biexp_ and ADC_B0-B5_	0.57**	0.56**	0.56**	0.54**	0.58**	0.54**	1

*ADC* monoexponential apparent diffusion coefficient, *D* diffusion coefficient derived from the biexponential model, *B0-B5* and *B0*_*biexp*_ b value combinations defined in Table 1, *deviation*

* *P* < 0.05, *deviation* ** *P* < 0.01 and *deviation* *** *P* < 0.001 (Wilcoxon signed rank test), Spearman’s *ρ* correlation test between median D_B0biexp_ and ADC_B0-B5_, ρ** *P* < 0.01

The median biexponential D* was 11.9 (range 4.3–72.1) and median f_p_ was 16 % (range 5–55 %). There was no correlation between D* and ADCs calculated using the three lowest b values ADC_B7–B8_.

## Discussion

This study shows that there is significant variability in monoexponentially calculated breast tumour ADCs when different b value combinations are used. However, for clinically used b value combinations (B0–B5), the maximum deviation is only 11.8 % (Table 3). The variability in calculated ADCs is reduced when low b values are omitted. Although ADCs are overestimated compared with biexponentially calculated Ds, there is a strong correlation between these two parameters.

Breast tumour ADCs for clinically used b value combinations in our study (mean 1.07–1.16 × 10^-3^ mm^2^/s, median 0.97–1.09 × 10^-3^ mm^2^/s) are within the range of previously reported values (0.68–1.61 × 10^-3^ mm^2^/s) [12, 13]. Consequently, for single-institution monitoring of individual treatment response using ADC, the b value combination may be of limited value, especially if low b values are excluded. But, clinical use of ADC cut-off values for tissue differentiation requires standardisation of acquisition protocol.

It has been suggested that the choice of b value combination have limited impact on the differentiation between malignant and benign breast lesions as studies have shown equal diagnostic performance of monoexponential flow-insensitive ADCs and ADCs calculated including low b values [12, 19, 20]. However, Ds from the biexponential model have been shown to provide better differentiation than ADCs [21]. Furthermore, including b values >1,000 s/mm^2^ in the ADC calculation might improve the differential diagnostic accuracy [11] as this reduces the contamination from microperfusion and ADC approaches the biexponentially derived D. But, if the b value is too high for the available signal-to-noise ratio the ADC will be underestimated. In our study, flow-insensitive ADCs of malignant breast lesions were correlated with biexponentially calculated Ds (*P* < 0.01), but overestimated by about 23 % (Table 3), which is in accordance with previously published results [21]. This may reflect the high vascularity and high cellularity of these lesions; further radiological–histopathological correlation analyses are needed. The microperfusion effect in normal breast tissue has been shown to be limited, yielding similar ADCs and Ds [21, 22], and thereby supporting the validity of the biexponential model.

A limitation to our study is the small and homogeneous study population, consisting of patients with large malignant tumours (mean 6.7 cm). Although our study included two different tumours types, i.e. invasive lobular (25 %) and ductal (75 %) carcinomas, there were no apparent differences between these types with respect to growth pattern, ADCs or contrast-enhancement characteristics on DCE MRI.

The large inter-patient variations in monoexponential ADCs calculated from only low b values found in this study may be attributed to microperfusion heterogeneity, a feature being of great importance in the exploration of anti-angiogenic treatment [23]. It has been hypothesised that D* and f_p_ may reflect microvessel perfusion. Weak correlations have been reported between f_p_ and initial enhancement on DCE MRI [21]. But in accordance with our results, the SDs of f_p_ and D* were large [21], and the clinical use of these parameters is questionable. The biological relevance of the biexponential model is not established as there is no clear evidence that the modelled compartments correspond to intravascular and extravascular flow and diffusion. Thus, other mathematical models may be more suitable for describing the DW MRI SI attenuation [24].

In our study the biexponential model provided poorer fitting than did the monoexponential model. This is probably a result of the limited number of low b values included. Since at least four DW MRI series with different b values are required in the biexponential model, the accuracy of the derived parameters is anticipated to increase with increasing number of b values. Despite the limited number of b values used in our study, calculated Ds, D*s and f_p_s are comparable to those obtained in a study using several more b values [21]. The selected number of b values causes a trade-off between increased precision in the estimated diffusion parameters and acquisition time.

In this study, median and mean diffusion values from single ROIs, placed within a solid part of the lesions, were used. This was done to minimise the influence of partial volume effects and limited spatial resolution. The differences between median and mean ADCs were found to be to the same order as the differences between ADCs calculated using the different b value combinations (B0–B5). Histogram analysis including the entire lesion would perhaps depict lesion heterogeneity better. However, mean values obtained from single ROIs have been used in previously published clinical DW MRI studies of breast cancer [2].

In conclusion, this study demonstrates that using different b value combinations strongly influences the calculated ADCs. Removing low b values reduces the perfusion contamination of the diffusion signal and ADCs approach the biexponentially calculated Ds. Thus, care should be taken when ADC calculation includes low b values. Future studies involving a larger number of patients should be undertaken in order to further validate these findings.
